# Radiotherapy for Thymic Carcinoma: Adjuvant, Inductive, and Definitive

**DOI:** 10.3389/fonc.2013.00330

**Published:** 2014-01-10

**Authors:** Ritsuko Komaki, Daniel R. Gomez

**Affiliations:** ^1^Department of Radiation Oncology, University of Texas MD Anderson Cancer Center, Houston, TX, USA

**Keywords:** postoperative radiation therapy, concurrent chemoradiation therapy, proton therapy, tumor histology, cardiac toxicity

## Abstract

Although historically thymoma and thymic carcinoma have been treated surgically, radiation therapy also has an important role, either as postoperative therapy to reduce the risk of mediastinal recurrence or as part of definitive treatment for patients who cannot undergo surgery. Induction chemotherapy and molecular targeted agents may also be appropriate for thymic carcinoma, the behavior of which resembles non-small-cell lung carcinoma more than that of thymoma or invasive thymoma and is increasingly being treated like lung cancer. We present here a review of current therapies for thymic malignancies and briefly discuss the potential benefits from novel technologies for such treatment.

Thymic malignancies, although the most common primary neoplasms of the anterior mediastinum, are relatively rare. The course of disease can range from indolent to quite aggressive, making the choice of treatment and analysis of outcomes challenging. The few prospective trials that have included patients with advanced-stage disease involved heterogeneous treatments (1). Retrospective and prospective analyses have demonstrated promising survival rates for patients undergoing trimodality therapy (chemotherapy, surgery, and radiation therapy) for advanced invasive thymoma (2–5) or thymic carcinoma (6–8). However, investigations that include large numbers of patients from several institutions are sparse, particularly those that compare patients who received different types of treatments.

## Indications for Adjuvant (Postoperative) Radiation Therapy

### R0 (completely resected) thymic malignancies

In general, radiation should be considered more strongly as the risk of recurrence increases. Therefore, for patients with the lowest likelihood of recurrence (i.e., completely resected Masaoka stage I thymoma), radiation can be safely omitted (9–11). For those at intermediate risk of local recurrence after complete resection, i.e., those with aggressive tumor histologies (such as thymic carcinoma) or Masaoka stage II and stage III disease, retrospective evidence exists both to support (9, 12) and contradict (13–15) claims of benefit from adjuvant radiotherapy after complete resection. In general, our institutional practice includes postoperative radiation for completely resected Masaoka-Koga stage III thymoma and stage II or III thymic carcinoma. Risk assessment and stratification is usually done in a multidisciplinary setting and drives the choice of adjuvant treatment. The International Thymic Malignancy Interest Group (ITMIG) published a set of definitions and reporting guidelines for the use of radiation therapy for thymic malignancies in 2011 (16). Pertinent recommendations for postoperative therapy are as follows. First, the term “postoperative” should be used for situations in which the tumor is resected and no residual disease is evident on imaging. If gross disease is present on postoperative imaging, then the disease should be defined as “recurrent” and the intent as “radiation for postoperative disease.” Second, the minimum acceptable dose for postoperative R0 disease is 50 Gy in 5 weeks. Finally, radiation to elective nodal regions is not recommended, and the extent of malignancy before surgery should be used as a guide for designing the treatment fields.

### Microscopic positive margins (R1) and gross disease (R2)

Radiation for R1 or R2 thymic malignancies should be started within 3 months of surgical resection. Doses between 40 and 64 Gy are most appropriate for microscopically positive margins, whereas doses of 54 Gy or higher should be used for gross disease; both should be given in standard fractions of 1.8–2.0 Gy (16). Patients with positive margins should be considered for concurrent chemotherapy and radiotherapy, especially patients with thymic carcinoma.

## Indications for Induction (Preoperative) Radiation Therapy

Some patients with thymoma and thymic carcinoma present with disease that is unresectable at diagnosis. Neoadjuvant chemotherapy can increase the likelihood of resectable disease for patients who are otherwise eligible for surgery. Potential induction regimens include chemotherapy alone (17, 18), radiation alone (19, 20), and combined chemotherapy and radiation (3). Chemotherapeutic regimens are generally cisplatin-based; regimens used in prior studies have included cyclophosphamide, doxorubicin, cisplatin, and prednisone or cisplatin and etoposide (18, 21). In some cases, patients undergo preoperative radiation therapy and then, after surgical resection, are considered for adjuvant treatment. Radiation therapy in this context can be difficult to deliver without exceeding dose constraints, and the cumulative radiation dose should be considered carefully. As is true for postoperative therapy, preoperative therapy should not include elective nodal regions but rather should encompass the gross area of involvement with an appropriate margin.

## Indications for Definitive Radiation Therapy

Definitive radiation therapy is generally used for patients who are not candidates for surgery because of either the extent of disease at diagnosis or other medical conditions. Because chemotherapy is a known radiation sensitizer, the combination of chemotherapy and radiation is considered most likely to control disease in these circumstances (5, 22–24). A multimodality approach such as trimodality therapy or definitive concurrent chemoradiation in unresectable cases is recommended for thymic carcinoma regardless of disease stage (25). Notably, only one-third of patients with thymic carcinoma are able to undergo a complete resection after such therapy (26). Definitive radiation therapy for unresectable disease is considered analogous to radiation therapy for disease that recurs after surgical resection; for such cases, we recommend radiation doses of 60–66 Gy to encompass gross disease plus a margin for microscopic regions at risk. The corresponding radiation dose when radiation is to be given with chemotherapy as definitive therapy is 54 Gy.

### Induction chemotherapy and molecular targeted therapies

Induction chemotherapy regimens for the treatment of thymic carcinoma and invasive thymoma have typically involved three cycles of cyclophosphamide, doxorubicin, cisplatin, and prednisone, to be followed by surgery. However, the advent of targeted therapies over the past decade has led to a variety of approaches being tested for their ability to improve disease control. Molecular characterization of thymomas and thymic carcinoma have shown that cKIT (CD117) is overexpressed in thymic carcinomas but not in thymomas (27, 28), and vascular endothelial growth factor (VEGF)-A and the VEGF receptor (VEGFR) are often overexpressed in thymic carcinomas (29). In contrast, mutations in the epithelial growth factor receptor are quite uncommon in thymic malignancies and seem to be limited to a small proportion of Asian patients (30, 31). These and other findings have led to several trials to test the safety and efficacy of targeted agents for thymic malignancies, including cKIT inhibitors, anti-EGFR therapies, and histone deacetylase inhibitors. To date, the results of these trials have been disappointing (30, 32). However, as more data on the molecular characteristics of thymic carcinoma are collected through multi-institutional tumor registries and databases, clinical trials of individualized care based on the molecular makeup of each individual tumor could lead to substantial breakthroughs in the treatment of this disease.

### Radiation techniques

Because of the central location of thymic malignancies and the relatively high doses used in radiation therapy, we strongly recommend the use of conformal techniques, such as three-dimensional conformal radiation therapy (3D-CRT), intensity-modulated radiation therapy (IMRT), or, if available, proton beam therapy because of the physical properties of the proton particles, which lead to lower doses being deposited both proximal and distal to the target volume. In addition, because tumors can show substantial changes in shape or size over the course of several weeks of radiation therapy, we recommend that when radiation is to be used as definitive therapy, adaptive replanning should be considered (33). Adaptive replanning involves obtaining additional CT scans at approximately halfway through treatment and evaluating tumor coverage to determine if the treatment plans should be modified. Several studies have shown that adaptive replanning can significantly minimize the radiation dose to normal tissues (34, 35).

For locally advanced disease, standard radiation treatment fields would include the tumor plus a margin if the radiation is to be given before surgery or as definitive therapy, and the tumor bed plus a margin if the radiation is to be given after surgery. Because lymph node metastases are rare, elective nodal coverage is not routinely recommended. Previous studies have examined the role of hemithoracic radiation therapy to prevent pleural dissemination. However, the doses that could be achieved while simultaneously meeting normal tissue constraints (10–20 Gy) were lower than are typically used for microscopic disease (36–38). Although this method has been reported to be safe and perhaps to lead to modest reductions in pleural recurrences, these findings are provocative at best, and these doses may well be ineffective for adequately controlling disease. The advent of conformal techniques, particularly IMRT, may enable the delivery of higher doses of radiation, which would increase the probability of disease control, while still limiting the dose to the surrounding esophagus, spinal cord, heart, and contralateral lung. Indeed, IMRT has been used to treat mesothelioma with promising results (39, 40).

## Long-Term Consequences of Radiation on the Heart and Vasculature

Abundant evidence suggests that long-term survivors of mediastinal radiation therapy are at risk of both acute and chronic cardiac sequelae. With regard to acute effects, the dose and fractionation of the radiation and the volume of heart irradiated all affect the risk of pericarditis and pericardial effusion (41). Given the close physiologic association between perfusion and ventilation, one might expect that radiation to the heart could affect lung function and vice versa. In one clinical study, investigators found that several heart dose-volume variables predicted radiation pneumonitis and that the fit of a model predicting pneumonitis was improved by the incorporation of cardiac variables (42). The risk of cardiac and other side effects is also affected by the exact location of the irradiated field. Short-term surrogates of long-term toxicity such as findings on cardiovascular imaging or biomarker correlates would be helpful for identifying which patients at greatest risk for cardiac events. In the meantime, we recommend the continued use of advanced radiation therapy technologies such as IMRT, proton beam therapy, use of 4D CT-based imaging and treatment planning, and adaptive planning when possible to minimize the dose to mediastinal structures for patients with thymic disease, many of whom will survive for several decades and thus will live to see the long-term consequences of irradiation of these vital organs.

## Effects of Radiation Therapy on Survival and Disease Control

Studies evaluating whether the inclusion of radiation therapy affects disease control and survival in thymic carcinoma are sparse because of the rarity of the disease; indeed, to the best of our knowledge no prospective trials have been done that compare outcomes between patients who have or have not received radiation therapy. Most published retrospective reports have focused on invasive thymoma, with some studies indicating that radiation is beneficial for locally advanced disease after surgery (8, 43–45) and others not demonstrating a clear advantage (46–48). Thymic carcinoma is often analyzed together with locally advanced invasive thymoma in an attempt to increase patient numbers and statistical power. In perhaps the best analysis of this type, a study of 1,320 patients from Japan with thymic malignancies, the authors compared adjuvant regimens in patients with completely resected stage III-IV thymoma and thymic carcinoma (14). They found no significant differences in survival between patients who received adjuvant radiation and those who received no further treatment. A subanalysis of only patients with thymic carcinoma also showed no difference in survival between those who received radiation versus no further treatment; however, that subanalysis did show that patients who received adjuvant chemoradiation for thymic carcinoma had worse outcomes than patients who received either radiation alone or observation. Notably, however, the size of these subgroups was small, with 24 receiving chemoradiation, 33 receiving radiation, and 16 receiving observation. A multivariate analysis of factors predicting survival among 290 patients with thymic carcinoma in the Surveillance, Epidemiology, and End Results database revealed that the extent of surgical excision, Masaoka stage, and race were associated with survival but receipt of radiation was not (49). In a much smaller study of 26 patients, investigators from Taiwan found that surgery and postoperative radiation achieved 5-year local control rates of 77%, but nearly 50% of patients experienced distant metastases (6). Finally, in a study of 14 patients with thymic carcinoma treated at the Gunma Prefectural Cancer Center in Japan, patients who received radiation had better outcomes than those who did not, and those investigators concluded that radiation has an important role in the treatment of thymic carcinoma (50). Clearly large prospective studies are needed to definitively answer this question.

As is true of studies of survival, analyses of patterns of failure after treatment for thymic carcinoma have generally been small and retrospective. In general, thymic carcinoma tends to recur distantly rather than locally, and pleural and extrathoracic metastases tend to drive overall survival (51, 52). For this reason, the development of effective targeted agents will be important in improving treatment outcomes in this disease. However, significant local invasion such as that into the great vessels or heart portends worse locoregional control and progression-free survival (53, 54). Further, both early-stage disease and achievement of complete resection have been shown to be associated with improved prognosis (55, 56), highlighting the role of radiation therapy in maximizing the likelihood of local control and optimizing survival outcomes.

## Conflict of Interest Statement

The authors declare that the research was conducted in the absence of any commercial or financial relationships that could be construed as a potential conflict of interest.
